# Case Report: 2-PAM or not 2-PAM

**DOI:** 10.5811/cpcem.39703

**Published:** 2025-04-07

**Authors:** Madelyn Huttner, Kahra Nix, Caitlyn Blair, Matthew Eisenstat

**Affiliations:** University of Louisville, School of Medicine Department of Emergency Medicine, Louisville, Kentucky

**Keywords:** acephate, organophosphate toxicity, pralidoxime, case report

## Abstract

**Introduction:**

Organophosphates (OP) are used as pest control agents worldwide and have been seen in accidental and intentional poisonings.

**Case Report:**

A patient presented after intentional ingestion of the OP Orthene (50% acephate). Due to copious secretions, the patient was intubated and given atropine by the paramedic before transport. In the emergency department he displayed both muscarinic and nicotinic effects from OP ingestion. The patient was given multiple doses of atropine and a pralidoxime bolus. He was extubated and transferred to psychiatry two days later.

**Conclusion:**

Acute OP exposure is a rare but complex presentation in the United States. In the United States there are bans on several organophosphate varieties, which have reduced the number and severity of OP toxicities. Acephate is generally considered a safer OP by United States regulators and the World Health Organization. In this case report, we describe an OP exposure with marked symptoms requiring intubation and successful treatment with atropine and pralidoxime. We also discuss the role of oximes in acephate toxicity.

## INTRODUCTION

Organophosphates (OP) are chemicals commonly used as pest control agents throughout the world. Organophosphate exposures have declined rapidly in the United States since the implementation of the Food Quality Protection Act in 1996.^1^ Although a rare occurrence in the US, emergency physicians should be aware of how to recognize and treat OP poisonings.

Orthene is a “fire ant killer” comprised of 50% acephate and a proprietary mixture. It is a fine powder that can easily be bought without license or regulation in the US at various stores and online. Acephate is a dimethyl OP with weak activity on the acetylcholinesterase enzyme.^2^ Acephate is generally considered a less toxic OP by the World Health Organization (Class II: Moderately Hazardous) and the US Environmental Protection Agency, which recently has proposed loosening environmental limits for the substance.^3^,^4^ A small number of case reports have been published describing acute acephate ingestions with severe cholinergic symptoms requiring atropine and oxime administration.^5^

## CASE REPORT

A 33-year-old, Burmese-speaking male patient presented to the emergency department (ED) by emergency medical services (EMS) after a neighbor called 9-1-1 upon finding a suicide note. His medical history was notable for a past suicide attempt. A witness noted that the patient had drunk an undetermined quantity of acephate in the form of the fire ant killer, Orthene. The EMS responders reported that the patient was fully oriented upon their arrival. However, he appeared diaphoretic, tachypneic, and was complaining of abdominal pain. Soon after their arrival, the patient’s mental status declined. He also developed copious secretions, vomiting, diarrhea, lacrimation, incontinence of urine, and spasms.

His blood glucose was 126 milligrams per deciliter (mg/dL) (reference range: 70–110 mg/dL). His initial vital signs on scene were as follows: blood pressure, 152/97 millimeters of mercury (mm Hg); heart rate, 93 beats per minute (bpm); respiratory rate, 22 breaths per minute; and oxygen saturation, 89% on room air. His temperature was not recorded. Intravenous (IV) access was obtained. Attempts to administer oxygen via nasal cannula and to suction secretions yielded no improvement in the patient’s respiratory status. The EMS responders progressed to administering 2 milligrams (mg) of IV atropine and intubated the patient. En route the patient received a total dosage of 4 mg of midazolam for agitation.

Upon arrival to the ED, the patient was increasingly agitated, necessitating 4 mg more of midazolam. His first vital signs in the ED were blood pressure 118/82 mm Hg, heart rate 101 bpm, and oxygen saturation 98% on the ventilator. His initial exam was notable for dry skin, clear lung fields bilaterally without secretions, sinus tachycardia, and no further diarrhea or lacrimation. However, he did have miosis and fasciculations. In conjunction with poison control the decision was made to infuse 2 grams of pralidoxime. He was also given an additional 2 mg of atropine IV. The hospital did not have additional pralidoxime, and the decision was made not to start an infusion as the patient had begun clinically improving rapidly. He was admitted to the intensive care unit and did not require any additional pralidoxime.

The patient’s serum laboratory evaluation was notable for glucose160 mg/dL; serum osmolality, 355 milliosmoles per kilogram (mOsm/kg) (reference range: 280–300 mOsm/kg); anion gap, 18 (2–11); serum bicarbonate, 18 millimoles per liter (mmol/L) (22–30 mmol/L); potassium, 2.3 mmol/L (3.7–5.0 mmol/L); lactate, 4.2 mmol/L (0.5–1.9 mmol/L); and creatine kinase, 274 units/L (U/L) (25–257 U/L). The serum drug screen resulted with an ethanol level of 169 mg/dL (0–10 mg/dL). The urine drug screen was positive for benzodiazepines, cocaine, and acetone. Red blood cell cholinesterase level was 8,273 international units/L (IU/L) (9,572–15,031 IU/L) shortly after presentation. Plasma cholinesterase level was 252 IU/L (3,334–7,031 IU/L).

On hospital day two, the patient was extubated after it was deemed that he had no evidence of continued central weakness and had not required additional atropine. He was observed in the intensive care unit for another 48 hours without incident and then transitioned to an inpatient psychiatric service.

## DISCUSSION

Organophosphate ingestion can be accidental or intentional. Exposure to OPs is far more common in developing countries and nations, particularly those with robust agricultural industries. Ingestion of OPs has long been a well-known and commonly used method of suicide in several Asian countries including Sri Lanka, India, and China.^6^ A combination of changing farming practices and regulations have led to decreased availability of OPs in the US; therefore, exposures presenting to the ED are rare.

**CPC-EM Capsule**

What do we already know about this clinical entity?

*Organophosphates (OP), like acephate, are used as pest control agents worldwide and have been seen in accidental and intentional poisonings*.

What makes this presentation of disease reportable?

*This case describing the successful management of an acephate ingestion is important as human toxicokinetic and toxicodynamic data for acephate are lacking*.

What is the major learning point?

*Despite its reported safety profile, acephate, an OP insecticide, can still result in severe presentations*.

How might this improve emergency medicine practice?

*In the United States, an OP toxidrome is a rare but complex presentation. This case describes the successful treatment of acephate toxicity with atropine and pralidoxime*.

Organophosphates competitively inhibit acetylcholinesterase (AChE) throughout the body. This prevents the hydrolysis and inactivation of acetylcholine (ACh), which leads to accumulation of ACh at synapses.^7^ Clinical signs from excess ACh are linked to the receptors involved, muscarinic and nicotinic.^7^ These symptoms include diarrhea, urination, miosis, bradycardia, bronchorrhea, bronchospasm, emesis, lacrimation, and salivation. The nicotinic symptoms involve mydriasis, tachycardia, hypertension, and seizures. Additional effects include fasciculations or weakness, with severe poisoning causing paralysis.^8^ This specific patient had many of the above-mentioned symptoms and fasciculations noted in the ED. Once OPs are bound to the active site of AChE, one of two processes occurs. Either the OP and AChE dissociate and the AChE recovers, or the OP forms a covalent bond with the AChE, permanently inactivating it. Recovery of AChE in OP-poisoned patients may take days to weeks.^7^

There is no laboratory test that will significantly alter management of OP toxicity from the ED. Red blood cell (RBC) and plasma cholinesterase levels can be obtained but will generally take days to return results. Plasma cholinesterase may drop faster in acute toxicity, but other disease processes can affect plasma cholinesterase results. A drop-in RBC cholinesterase activity is more specific for OP toxicity and correlates better with clinical weakness but may take longer to become apparent following acute poisoning. Our patient had a relatively normal RBC cholinesterase with a significantly low plasma cholinesterase (less than one-tenth reference range), which was likely due to early timing of labs in the setting of an acute OP toxicity.

Management of OP toxicity involves treating both the muscarinic and nicotinic effects. Decontamination is critical. Clothes with OP on them should be removed immediately, and patients should be washed with soap and water. Emesis from OP-poisoned patients should be contained, covered, and separated from healthcare workers as ingested OP can “off gas” from the emesis.^10^ Antidotal therapy includes atropine and an oxime.^8^ In the US, the typical oxime used is pralidoxime (2-PAM). Pralidoxime works by reactivating inhibited AChE that has not completed the aging process and should be initiated as soon as there is suspicion of OP ingestion.^11^ The rate of aging for each OP is different and varies widely. The time to aging for acephate is not known.

Pralidoxime given after the aging process will be ineffective.^11^ A typical loading dose is one to two grams intravenously (20–50 mg/kg in pediatrics) bolus over 15–30 minutes with various strategies of intermittent or continuous dosing for continued weakness. Pediatric continuous infusion recommended at 10–20mg/kg/hour per the FDA however controversy exists regarding optimal adult infusion dosing.^12^ Oximes may provide alternate benefits beyond preventing the aging process. They also may help reduce nicotinic symptoms and reduce the total dose of atropine required for stabilization. Controversy exists about the utility of 2-PAM and oximes in general in the treatment of OP toxicity. Our patient received only a loading dose at two grams with no infusion. His weakness and altered mental status improved on day two without additional atropine or oxime. He also had no delayed weakness, which is sometimes attributed to underdosing oximes. We suspect that pralidoxime did not make a significant impact on his recovery time since the dose was too low based on prior studies.^13^ That would mean the acephate probably dissociated from the AChE, and he recovered spontaneously. It is also possible that the aging for acephate is so weak that even an inadequate dose was able to interrupt the process. Regardless, not enough is known about acephate to determine whether oximes play a major role in acephate ingestions.

## CONCLUSION

Human toxicokinetic and toxicodynamic data for acephate is lacking. The overall chemical profile of acephate suggests a safer product, but we report a severe presentation of acephate toxicity treated successfully with atropine and pralidoxime. It is important for physicians to know that acephate, despite its safety profile, can still result in severe presentations. Emergency physicians are rarely exposed to OP-toxicity patients in the US and should be vigilant for patients presenting with symptoms of a cholinergic toxidrome. The need for oximes in the management of acephate toxicity is questionable and needs further study.
